# Prevention and infection control programs related to health assistance: diagnosis of hospitals in the state of Paraná, Brazil

**DOI:** 10.1186/2047-2994-4-S1-P273

**Published:** 2015-06-16

**Authors:** DCI Alves, V de Brito Poveda, RA Lacerda

**Affiliations:** 1 Medical-Surgical, Nursing Department, Cascavel, Brazil; 2 Medical-Surgical, University of São Paulo, São Paulo, Brazil

## Introduction

Prevention and Infection control programs associated with health assistance (PCIRAS), besides the contribution to reduce the number of these infections, build up relevant components of quality evaluation system from health assistance.

## Objectives

To characterize the performance of these programs in hospitals in the state of Paraná-Brazil.

## Methods

Prospective and transversal study of processual evaluation, through an instrument previously validated, built up by four indicators whose contents are the expected development of these programs in relation to Brazilian and international literature requirements. The indicators are: 1) Technical operational structure (TOS); 2) Operational Guidelines (OGS); 3) Epidemiological Surveillance System (ESS); 4) Control and Prevention Proceedings (CPP). The study was performed from 2013 to 2014 in 50 hospitals statistically defined by access.

## Results

The general conformity obtained by these programs were 71,0%, with dispersion (dp) of 23,88. The conformities of each indicator were: TOS - 79,4% and 18,9dp; ESS – 76,0% and 30.5dp; OGS - 65,5% and 26,9dp; CPP - 63,2%/39,5dp. The general development was a bit below those previously expected (75%), due to OGS and CPP indicators. The programs presented minimum data to be operated and to provide epidemiologic observation, but then it is damaged due to quantitative and qualitative insufficience of operational and action norms to the control and prevention of these infections. The presence of health quality certification, internal fiscalization control, presence of an exclusive nurse to work on the Hospital Infection Control Service (HICS), presence of contracted and state physicians, longer hours of exclusive dedication and wider experience of physicians and nurses, present great association to improve the development of 11 CPIRAH, respectively.

## Conclusion

As Paraná is one of the most developed states in Brazil, the result of this study is something to be worried about. It motivates the necessity to recognize and characterize these programs in other regions of Brazil.

## Disclosure of interest

None declared.

